# Phenology and parasitism rates of *Xenos oxyodontes* and *Xenos moutoni* (Strepsiptera: Xenidae) in *Vespa* (Hymenoptera: Vespidae) in South Korea

**DOI:** 10.1051/parasite/2026028

**Published:** 2026-05-19

**Authors:** Jaehee Kim, Chang-Jun Kim, Moon Bo Choi, Ohseok Kwon

**Affiliations:** 1 Department of Applied Biology, Kyungpook National University Daegu Republic of Korea; 2 Division of Forest Biodiversity, Korea National Arboretum Pocheon Republic of Korea; 3 Institute of Agricultural Science and Technology, Kyungpook National University Daegu Republic of Korea; 4 Department of Plant Medicine, College of Agriculture and Life Sciences, Kyungpook National University Daegu Republic of Korea

**Keywords:** Host association, Voltinism, Developmental stage, Parasitism

## Abstract

The long-term seasonal parasitism patterns, hereafter referred to as stylopization, and within-nest developmental stage distributions of *Vespa*-associated Xenidae (Strepsiptera) remain poorly characterized in South Korea. We compiled 15 years of data (2008–2023) based on trapped *Vespa* individuals from multiple regions and quantified stylopization rates for two Xenidae species, *Xenos oxyodontes* and *Xenos moutoni*, based on diagnostic characters visible on stylopized hosts. Seasonal occurrence was summarized primarily from female *Xenos* specimens, which can be identified to species, and host-association patterns were assessed where possible. To complement the trap-based records, we dissected 22 *Vespa analis* colonies collected in September 2020 and documented strepsipteran developmental stages within the nests. Across 39,610 examined host individuals, stylopization rates and seasonal occurrence differed between the two *Xenos* species and among host taxa, indicating distinct host-use and temporal activity patterns. Importantly, the nest dissections provided direct observations of late-season developmental stage compositions, enabling us to compare within-nest reproductive/developmental stages to those inferred from trap-detected stylopized hosts. This comparison revealed that key reproductive-stage information may not be captured by trap records alone. Thus, integrating long-term trap data with nest-level observations can clarify species-specific phenology and developmental/reproductive stage distributions in *Vespa*-associated Xenidae, providing a better empirical context for interpreting stylopization intensity and developmental timing in field populations.

## Abstract (fr)

Les schémas de parasitisme saisonnier à long terme, désignés ici par le terme de stylopisation, et la distribution des stades de développement au sein du nid des Xenidae (Strepsiptera) associés à *Vespa* restent mal connus en Corée du Sud. Nous avons compilé des données sur 15 ans (2008–2023) à partir d’individus de *Vespa* piégés dans plusieurs régions et quantifié les taux de stylopisation pour deux espèces de Xenidae, *Xenos oxyodontes* et *Xenos moutoni*, en nous basant sur les caractères diagnostiques visibles chez les hôtes stylopisés. L’occurrence saisonnière a été résumée principalement à partir de spécimens femelles de *Xenos*, qui peuvent être identifiés jusqu’à l’espèce, et les schémas d’association hôte-parasite ont été évalués lorsque cela était possible. Pour compléter les données de piégeage, nous avons disséqué 22 colonies de *Vespa analis* collectées en septembre 2020 et documenté les stades de développement des Strepsiptères au sein des nids. Sur 39 610 individus hôtes examinés, les taux de stylopisation et la fréquence saisonnière différaient entre les deux espèces de Xenidae et entre les taxons hôtes, indiquant des profils d’utilisation de l’hôte et d’activité temporelle distincts. Surtout, la dissection des nids a fourni des observations directes de la composition des stades de développement en fin de saison, nous permettant de comparer les stades de reproduction et de développement au sein des nids à ceux déduits des hôtes stylopisés détectés par piégeage. Cette comparaison a révélé que des informations clés sur les stades de reproduction ne sont pas nécessairement accessibles par les seuls relevés de piégeage. Ainsi, l’intégration de données de piégeage à long terme avec des observations au niveau des nids peut clarifier la phénologie spécifique à l’espèce et la distribution des stades de développement et de reproduction chez les Xenidae associés à *Vespa*, fournissant un meilleur contexte empirique pour interpréter l’intensité de la stylopisation et le calendrier de développement dans les populations naturelles.

## Introduction

Species of the order Strepsiptera are obligate endoparasites of other insects, and they are well known for their extreme sexual dimorphism and highly specialized reproductive biology [12, 13, 17, 31]. In all but the primitive members of Mengenillidae, females exhibit neoteny and remain larviform within the host, where they act as parasites and reproduce, whereas males emerge, becoming free-living adults, and are typically short-lived. After reaching maturity inside the host, the female exposes only the cephalothorax through the host’s abdominal integument, and mating occurs at this exposed region. The female then larviposits triungulins. These triungulins represent the dispersal stage; they leave the original host, move to a new host, and enter its body, where development continues [2, 8, 14, 16, 28, 31]. Given this life history, strepsipteran parasitism is best viewed as a long-term interaction that is tightly coupled to the host’s survival and seasonal activity.

Specialized strepsipteran parasitism is commonly referred to as stylopization, and a wide range of physiological and behavioral alterations have been documented in stylopized hosts [1, 2]. Such effects have been discussed with particular frequency for species in the strepsipteran family Xenidae, which parasitize eusocial vespid wasps. Among vespids, the *Polistes*–*Xenos* association is the most well-studied, with reported effects including host sterilization, the suppression of ovarian development, the cessation of worker tasks, nest abandonment, and extranidal aggregation [9, 10, 18, 27]. Transcriptomic evidence further suggests that in *Polistes*, stylopization can be accompanied by changes in gene-expression patterns in the brain [2, 6], raising the possibility that transcriptomic profiles associated with the worker caste are attenuated and remodeled toward a more gyne-like state. Such shifts also appear to be associated with extended worker longevity. Mature strepsipteran females can induce diapause in workers after mating [4, 34], and larviposition has been reported to occur the following spring after the host terminates diapause [23, 25, 30, 34].

Outside of the *Polistes*–*Xenos* system, basic ecological information on many *Vespa*–*Xenos* associations, including host specificity and seasonal developmental patterns, remains relatively limited, despite the existence of observational records. Among *Vespa*-associated *Xenos* species, *Xenos oxyodontes* Nakase & Kato, 2013 and *Xenos moutoni* Buysson, 1903 are the most commonly referenced. They were previously treated as the same species, *X. moutoni* [22, 24], but were recently redescribed as distinct cryptic species [29]. The two species are generally considered to differ in host specificity. Stylopization by *X. oxyodontes* has primarily been recorded in *Vespa analis* Fabricius, 1775, with additional cases reported from *Vespa simillima* Smith, 1868 and *Vespa velutina* de Lepeletier, 1836, whereas stylopization by *X. moutoni* has been observed in multiple *Vespa* species, but not *V. analis* [11, 15, 29]. This distinction suggests that species-level resolution within *Xenos* is necessary when interpreting phenology and parasitism ecology. In Japan, studies have quantified seasonal occurrence patterns and parasitism levels for *X. moutoni* and *X. oxyodontes*, providing an important baseline for interpreting field observations of *Vespa*-associated *Xenos* species [21, 23, 25, 30]. However, while trap-based data are useful for describing seasonal occurrence, they cannot directly show where individuals transition into the developmental and reproductive stages. Thus, empirical data regarding whether *Vespa*-associated *Xenos* mate within or outside of the host’s nest are scarce. To acquire such data, nest dissection can be used to characterize patterns of mature parasite stages within the nest and to formulate hypotheses on the spatial context of reproductive events [23].

Our study aimed to provide a baseline synthesis of the phenology and stylopization patterns of two Xenidae species, *X. moutoni* and *X. oxyodontes*, in *Vespa* using material collected in South Korea. First, using multi-year, multi-region trap data, we examined seasonal occurrence patterns and geographic distributions based primarily on female specimens, which can readily be identified to species, and we also assessed host-association patterns where possible. In addition, to evaluate the distribution of developmental and reproductive stages of *Xenos*, information not readily inferable from trap data alone, we used stylopized hosts collected from *V. analis* nests to characterize the reproductive-stage distributions of female and male *X. oxyodontes* individuals within hornet colonies. By integrating the broad-scale temporal patterns provided by trap-based records with the stage-specific information obtained from colony samples, we refined the baseline knowledge on when, where, and during which stages stylopization occurs in hornets, and, within the limits of our data, we make conservative inferences regarding expected local stylopization intensities and voltinism.

## Materials and methods

### Specimen collection

#### Nest collection

On September 2, 2020, we collected 22 active *V. analis* colonies from Muui Island (Jung-gu, Incheon Metropolitan City, South Korea). For each colony, recovered individuals were preserved in 99% ethanol, and we recorded the number of individuals by caste (queen, worker, and male).

#### Trapping

**Figure 1:**
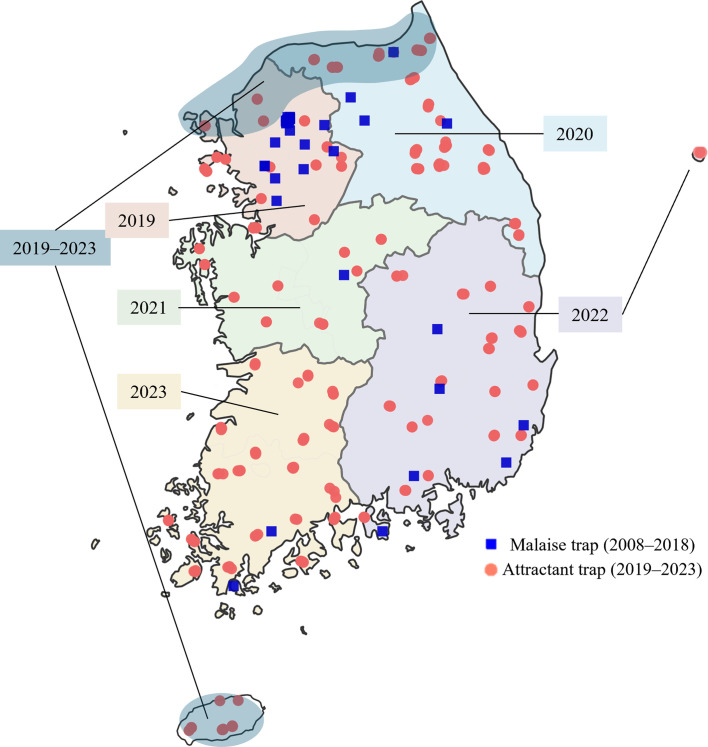
Spatial distribution of 677 trapping sites in South Korea (2008–2023). Blue squares represent Malaise trap sites, sampled from 2008 to 2018, and red circles indicate sugar-attractant trap sites, sampled from 2019 to 2023. Shaded areas approximate the year-specific sugar attractant trap survey coverage over the 2019–2023 period, with the years specified on the map.

Trap surveys were conducted from 2008 to 2023. In Incheon and Gyeonggi-do, traps were fixed at the same sites for multiple years, with additional traps installed in other regions during specific years. A total of 677 traps comprising a mixture of hornet attractant traps and Malaise traps were deployed (Fig. 1, Supplementary file). Traps were installed each year in May, and insects were retrieved at approximately one-month intervals until trap removal in November, and a single “visit,” as used in the statistical analyses, was defined as one trap-check and collection event. From the collected samples, *Vespa* individuals were sorted and preserved in 99% ethanol.

### Specimen examination and classification

**Figure 2:**
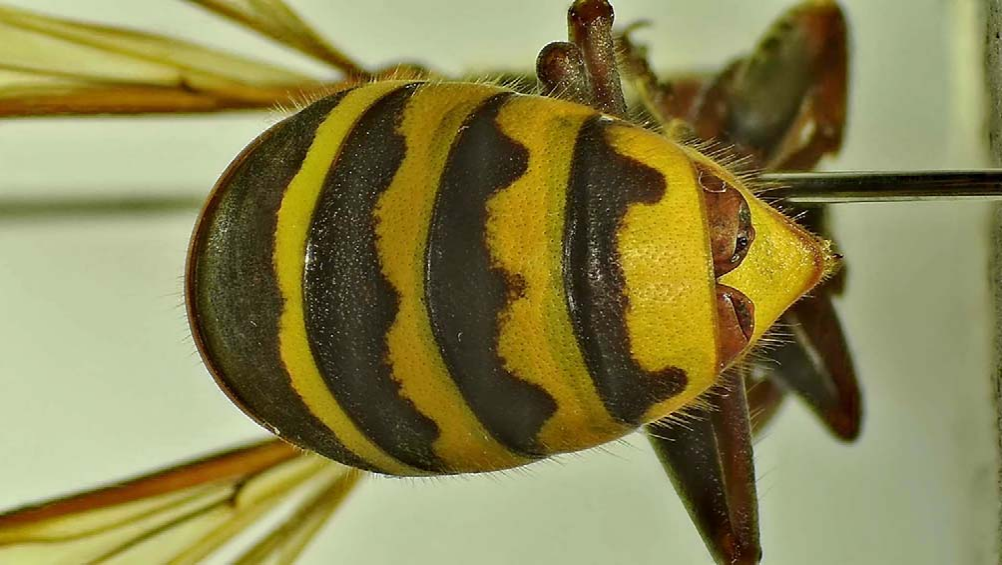
A female *Xenos moutoni* stylopizing a host hornet, *Vespa crabro*, protruding from the host abdomen.

All specimens were examined under a stereomicroscope, and individuals with a strepsipteran cephalothorax protruding between the abdominal tergites were confirmed as stylopized hosts (Fig. 2). Stylopized hornets were stored individually, and we recorded *Vespa* species and caste and *Xenos* species and sex (based on the female cephalothorax or traces of male puparium/adult emergence). Female caste was distinguished based on relative body size [27]. *Xenos* were identified following Nakase and Kato (2013) [29].

*Xenos* individuals were assigned to stages based on sex-specific reproductive characteristics: females were classified as pre-vitellogenic, vitellogenic, gravid, larvipositing, or post-larviposition, and males were classified as pupa, transitional, not emerged, or emerged (Table 1). For trap samples, since installation and collection dates differed among traps, we used the midpoint date between trap installation and collection as the capture timing for each specimen to minimize error.

**Table 1:** Diagnostic criteria for reproductive and developmental stage assignment in *Xenos* spp.

Sex	Stage	Diagnosis
Female	Pre-vitellogenic	Internal structures intact; oocytes absent or poorly developed.
	Vitellogenic	Body cavity filled with well-developed oocytes.
	Gravid	Fertilized eggs and developing embryos and/or triungulins present.
	Larviposition	Triungulins concentrated toward the cephalothorax and actively exiting via the birth opening (brood canal).
	Post-larviposition	Most triungulins expelled; few or none remaining in the body.
Male	Pupa	Pupa present within puparium.
	Transitional	Late pupa to soft, unsclerotized near-adult retained within the puparium.
	Not emerged	Pharate adult retained within puparium; no emergence.
	Emerged	Puparium empty; emergence opening/exuviae indicates adult emergence.

### Statistical analysis

All statistical analyses and visualizations were conducted in R (4.4.2).

#### Phenology analysis

To evaluate differences in female *Xenos* phenology among reproductive stages, we compared the distributions of capture times (midpoint date) across stages. Because the data did not meet the assumption of normality and sample sizes among stages were unbalanced, we used a Kruskal–Wallis test. When a significant difference was detected, we conducted *post hoc* pairwise comparisons using Dunn’s test with the Holm correction for multiple comparisons. We calculated the effect size (*ε*^2^) as an index of the extent to which stage membership explains variation in midpoint capture time:

$$\varepsilon^{2}=\frac{H-k+1}{n-k},$$

*H*: test statistic

*k*: number of groups

To maintain consistency in sample sizes among stages, we pooled pre-vitellogenic and vitellogenic females into a single group and pooled gravid and larvipositing females into a single group, yielding three reproductive-stage groups for day of year (DOY) distribution comparisons.

#### Distributional analysis

Trap-based surveys are subject to spatial capture bias, such that in some areas, species may be present but, nevertheless, go undetected [32]. For eusocial wasps, in particular, attraction to sugar-based baits varies with bait type and resource responsiveness, and trap efficiency can therefore differ markedly across species and environmental contexts [20, 26]. In our analysis, the response variable was defined as the number of stylopized hosts per trap visit for each trap ($Y_{i}$). To estimate the trap-level intensity of *Xenos* stylopization, we fitted a negative binomial generalized linear model to $Y_{i}$ using a log link, including the total number of host individuals captured during the same trap visit (*Hᵢ*) as an offset. This analysis follows Heusler *et al.* (2025) [7] and is not intended to infer the causal effects of environmental covariates, but to summarize where and to what extent host-capture–adjusted expected stylopized-host events are spatially concentrated within a consistent spatial sampling framework.

$$Y_{i}\sim \text{NegBin}\left(\mu_{i},\theta \right),$$

$$\ln\left(\mu_{i}\right)=\beta_{0}+\ln\left(H_{i}\right).$$

Here, $\mu_{i}=\exp\left(\beta_{0}\right)H_{i}$ represents the expected number of stylopization events, with $\exp\left(\beta_{0}\right)$ corresponding to the mean stylopization event rate per host individual; *θ* is the overdispersion parameter of the negative binomial distribution; and *H_i_* denotes the number of potential host individuals captured during the same trap visit, with in $\left(H_{i}\right)$ included as an offset to adjust for differences in host captures among trap visits. We used the estimated *μ_i_* value as the expected stylopized host count for each trap.

For the spatial analysis of *X. moutoni*, we included all traps in which hornets were captured as analysis units, reflecting the strepsipteran species’ relatively broad host spectrum among *Vespa* species. In contrast, *X. oxyodontes* is more host-specific, and traps without captures of its primary hosts, *V. analis* and *V. simillima simillima*, provide no opportunity for stylopization estimation. Accordingly, for the spatial analysis of *X. oxyodontes*, we restricted the analysis to traps that captured at least one *V. analis* or mainland *V. simillima* individual, excluding structural zeros arising from host absence. Therefore, the results should be interpreted as variation in stylopization intensity conditional on host detection. Although *X. oxyodontes* has occasionally been reported from *V. velutina*, no stylopized individuals were detected in this host in our trap samples. Therefore, traps capturing only *V. velutina* were not included as potential host records in the distribution analysis.

To visualize spatial patterns, we applied hexagonal binning based on trap coordinates. Within each hexagon, we calculated the mean of $\mu_{j}$ across all visits to traps contained within that cell (${\hat{\mu }}_{h}$) to obtain a spatially aggregated expected stylopized-host count. This aggregation was used to smooth local variability at the individual-trap level and to summarize broad-scale spatial patterns. To compare the spatial concentrations of $\hat{\mu }$ values between *X. moutoni* and *X. oxyodontes*, we ranked hexagons in descending order of $\hat{\mu }$ and then calculated the cumulative proportion of $\hat{\mu }$ represented by each species as a function of the cumulative proportion of hexagons, constructing rank–cumulative curves. Concentration was quantified in two ways, as the proportion of the total cumulative $\hat{\mu }$ values accounted for by the top 10% of the hexagons, and by the area under the rank–cumulative curve (AUC). We defined AUC as a concentration index that quantifies deviation of the rank–cumulative curve from the uniform reference line (the diagonal) and interpreted larger AUC values as indicating a stronger concentration of $\hat{\mu }$ values in a smaller number of hexagons.

To assess whether the observed concentration metrics deviated from a random distribution, we conducted a permutation test. For each species, we randomly reassigned the $\hat{\mu }$ values across hexagon IDs and recalculated the concentration metrics, repeating this procedure 10,000 times to generate a null distribution. The *p*-value was then computed as the proportion of permutation runs in which the concentration metric was equal to or more extreme than the observed value.

Because our model included only an intercept and host availability, the spatial pattern of $\hat{\mu }$ primarily reflects how host captures are distributed within the sampling frame rather than a spatial effect of the parasite itself. Accordingly, we interpreted the results of the analysis described in this section as the spatial concentration of expected stylopization events under a consistent survey design, rather than as spatial heterogeneity in parasitism rate. Since no stylopization was observed in *Vespa* individuals captured in the two major island regions, Jeju Island and Ulleung Island (Table 2), these records were excluded from the distribution analysis.

**Table 2:** Number and stylopization status of *Vespa* individuals collected in traps across South Korea over fifteen years, organized by *Vespa* and *Xenos* species.

*Vespa* species (host)	Collected hornets	*Xenos* species	Stylopized hornets	Stylopization rate (%)
*V. analis*	1,747	*X. oxyodontes*	58	3.320
*V. simillima* (Mainland)	8,921	*X. oxyodontes*	2	0.022
		*X. moutoni*	6	0.067
*V. simillima* (Ulleung Island)	3,973	NA	0	0.000
*V. simillima* (Jeju Island)	1,943	NA	0	0.000
*V. velutina*	4,302	NA	0	0.000
*V. mandarinia*	1,950	*X. moutoni*	36	1.846
*V. crabro*	9,748	*X. moutoni*	50	0.513
*V. ducalis*	3,289	*X. moutoni*	27	0.821
*V. binghami*	2,011	*X. moutoni*	2	0.100
*V. dybowskii*	1,726	NA	0	0.000
Sum	39,610		181	
Stylopization rate (%) = (stylopized hornets) / (collected hornets) × 100				

## Results

A total of 39,610 individuals representing eight host species were collected, of which 181 were confirmed as stylopized hosts (Table 2). Among host species, *Vespa crabro* was the most frequently collected (9,748 individuals), whereas *Vespa dybowskii* was the least collected (1,726 individuals), and no stylopized individuals were detected for this species (0%). Additionally, no stylopized *V. velutina* individuals were detected, despite 4,302 individuals being collected (0%). The largest number of stylopized hosts was recorded for *V. analis* (58 individuals; 3.320%), followed by *V. crabro* (50 individuals; 0.513%), and *Vespa mandarinia* (36 individuals; 1.846%). The lowest rate was observed for *V. simillima* (Mainland), in which *X. oxyodontes* was detected at 0.022%. Notably, females in the larviposition stage were recorded in trap captures during July–September and also in October, and many of these records were from worker hosts. No stylopization was observed in *Vespa* captured on Jeju Island and Ulleung Island, the two major island regions.

### Phenology

**Figure 3:**
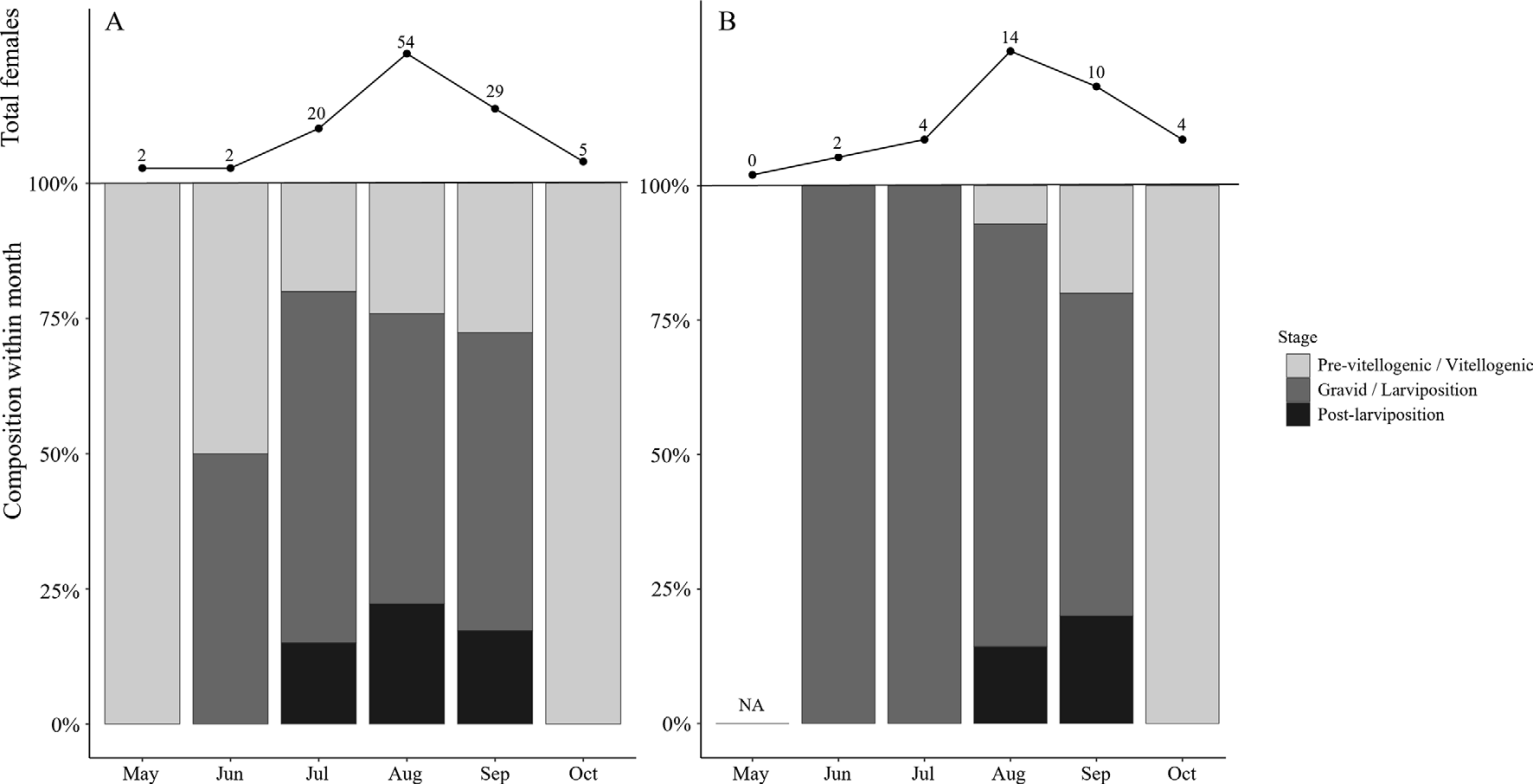
Monthly life-stage compositions of female (A) *Xenos moutoni* and (B) *Xenos oxyodontes* individuals captured by traps in South Korea. The lines (top) indicate the monthly number of captured females (*n*), and the stacked bars (bottom) show the within-month proportional compositions of female reproductive stages.

In *X. moutoni*, no significant difference among stages was detected (*H* = 0.405, *df* = 2, *p* = 0.817) (Fig. 3A). The pre-vitellogenic/vitellogenic stage had a median DOY of 230 (IQR of 220–253), the gravid/larvipositing stage had a median DOY of 224 (IQR of 217–244), and the post-larviposition stage had a median DOY of 224 (IQR of 217–240). Since stage differences were not significant in the Kruskal–Wallis test, we did not further interpret stage-specific differences for *X. moutoni*.

In contrast, significant differences among stages were detected in *X. oxyodontes* (*H* = 12.6, *df* = 2, *p* = 0.00182) (Fig. 3B). Dunn’s *post hoc* test with the Holm adjustment indicated that the pre-vitellogenic/vitellogenic and gravid/larvipositing stages were significantly different (*p*_adj = 0.00162), whereas other pairwise comparisons were not. The effect size for *X. oxyodontes* was *ε*^2^ = 0.342, indicating stage-associated differences in capture timing. The pre-vitellogenic/vitellogenic stage had a median DOY of 284 (IQR of 262–294), the gravid/larvipositing stage had a median DOY of 224 (IQR of 214–239), and the post-larviposition stage had a median DOY of 244 (IQR of 240–248). For *X. oxyodontes*, individuals in the gravid/larvipositing stage tended to be captured earlier in the year than those in the pre-vitellogenic/vitellogenic stage, and the overall effect of stage was primarily driven by the separation between these two stages.

### Distribution

**Figure 4:**
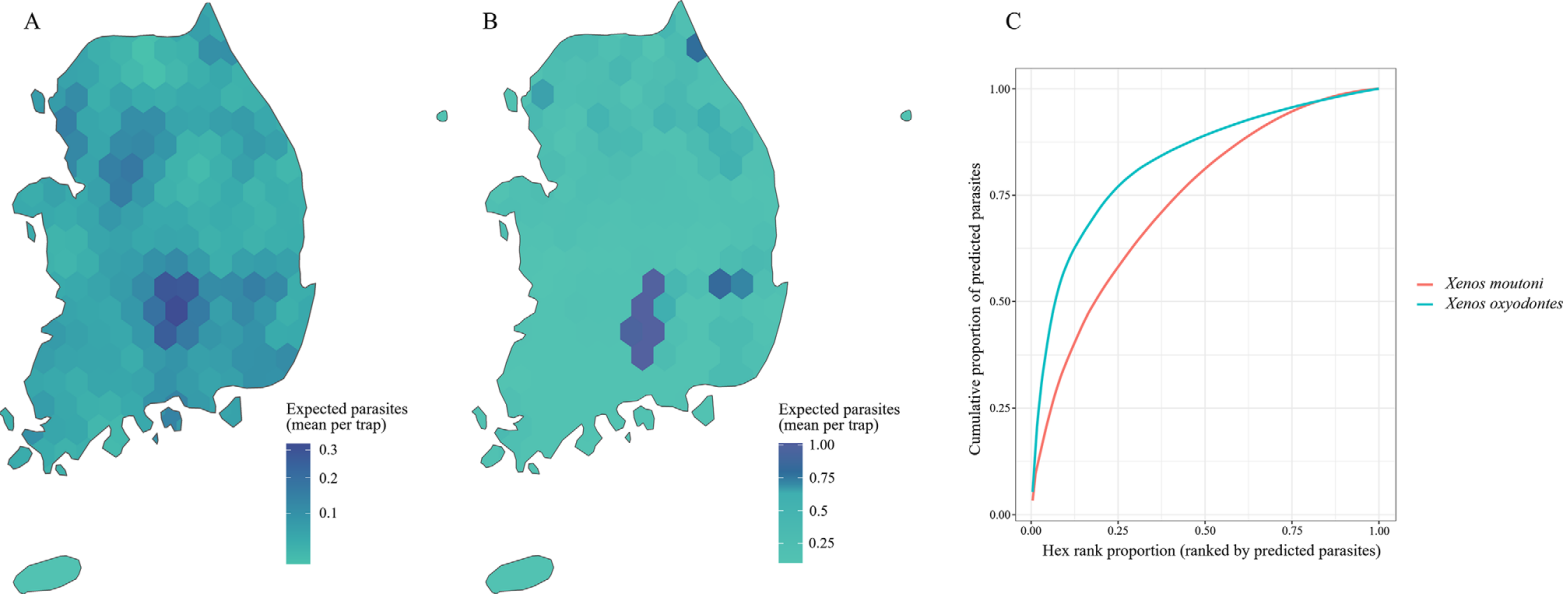
Spatial distributions of the GLM-based, host-capture–adjusted expected stylopized-host counts ($\hat{\mu }$) for two *Xenos* species, (A) *X. moutoni* and (B) *X. oxyodontes*, in South Korea. The expected numbers of stylopized hosts per trap were estimated using a negative binomial GLM and then binned into a hexagonal grid. (C) Rank–cumulative curves for each *Xenos* species showing the cumulative proportion of the total expected stylopized-host count ($\hat{\mu }$) across hexagons ranked in descending order of the expected total stylopized-host count. The spatial distribution patterns of the two species were significantly different based on permutation tests comparing their AUC values (*p* < 0.001).

To analyze the distributions of both *Xenos* species, we characterized the spatial concentrations of host-capture–adjusted expected stylopized-host counts ($\hat{\mu }$) by applying a consistent sampling frame and fitting a negative binomial GLM with an offset for host captures to the trap data. High $\hat{\mu }$-value hexagons tended to be more locally concentrated within the sampling frame for *X. oxyodontes* than for *X. moutoni* (Fig. 4). The top 10% of hexagons accounted for 23.94% of the total expected stylopized-host counts in *X. moutoni*, whereas the top 10% of hexagons accounted for 58.08% in *X. oxyodontes* (Figs. 4A, 4B), and the rank-curve–based concentration metric (AUC) was higher in *X. oxyodontes* (AUC = 0.827 for *X. oxyodontes* vs AUC = 0.736 for *X. moutoni*), further supporting our finding that this species’ expected stylopization events are more locally concentrated. Permutation tests indicated that the observed concentration metrics deviated significantly from those of the random-reassignment distributions (Fig. 4C). Of note, because the spatial summary for *X. oxyodontes* is conditional on trap visits after excluding structural zeros (*i.e.*, traps in which the primary hosts were not observed), this finding should be interpreted as the concentration of expected events within the sampling frame given host detection.

### Nest-level patterns

**Figure 5:**
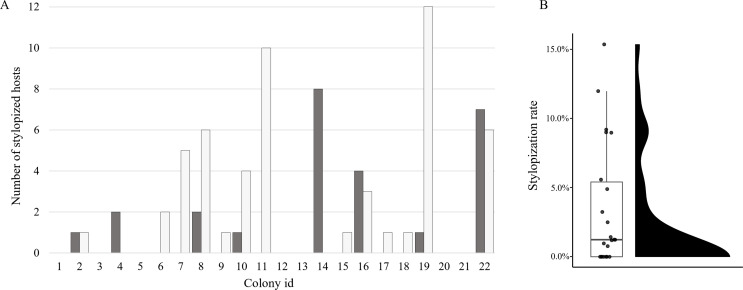
(A) Number of *V. analis* individuals stylopized by *X. oxyodontes* in each of the 22 colonies examined. Dark grey bars represent females, and white bars represent males. (B) Colony-level stylopization rate of *V. analis* by *X. oxyodontes*, calculated as the proportion of stylopized individuals among all adults examined in each nest. Each dot represents one *V. analis* colony, and the raincloud plot shows the distribution of colony-level stylopization rates.

Among 22 *V. analis* nests collected on Muui-do, Incheon (Table 3, Fig. 5), a total of 2,155 hornet individuals were recorded, of which 75 were parasitized. The mean number of hornet individuals per nest was approximately 98 (2,155/22), and the mean number of parasitized individuals per nest was approximately 3.41 (75/22). Nest-level stylopization rates showed substantial variation, ranging from 0% to 15.385%. The highest stylopization rate was observed in Nest 14 (15.385%), followed by Nest 22 (12.000%). In contrast, seven nests contained no stylopized individuals.

**Table 3:** Colony composition and stylopization in 22 *V. analis* nests collected from Muui-do, Incheon, South Korea on September 2, 2020.

Nest ID	Workers	Queens and gynes	Males	Total hornets	Stylopized hornets	Stylopization rate (%)
1	65 (0)	1 (0)	26 (0)	92 (0)	0	0.000
2	154 (1)	5 (0)	48 (1)	207 (2)	2	0.966
3	56 (0)	3 (0)	27 (0)	86 (0)	0	0.000
4	54 (2)	3 (0)	110 (0)	167 (2)	2	1.198
5	89 (0)	1 (0)	26 (0)	116 (0)	0	0.000
6	43 (0)	1 (0)	117 (2)	161 (2)	2	1.242
7	64 (0)	3 (0)	87 (5)	154 (5)	5	3.247
8	41 (2)	1 (0)	47 (6)	89 (8)	8	8.989
9	25 (0)	1 (0)	14 (1)	40 (1)	1	2.500
10	43 (1)	1 (0)	58 (4)	102 (5)	5	4.902
11	80 (0)	1 (0)	80 (9)	161 (9)	9	5.590
12	49 (0)	1 (0)	10 (0)	60 (0)	0	0.000
13	36 (0)	1 (0)	6 (0)	43 (0)	0	0.000
14	38 (8)	1 (0)	13 (0)	52 (8)	8	15.385
15	66 (0)	6 (0)	58 (1)	130 (1)	1	0.769
16	53 (4)	3 (0)	20 (3)	76 (7)	7	9.211
17	9 (0)	1 (0)	71 (1)	81 (1)	1	1.235
18	34 (0)	1 (0)	35 (1)	70 (1)	1	1.429
19	69 (1)	1 (0)	52 (10)	122 (11)	11	9.016
20	16 (0)	1 (0)	5 (0)	22 (0)	0	0.000
21	18 (0)	1 (0)	5 (0)	24 (0)	0	0.000
22	33 (6)	3 (0)	64 (6)	100 (12)	12	12.000

Numbers in parentheses indicate the number of stylopized individuals within each caste.

**Figure 6:**
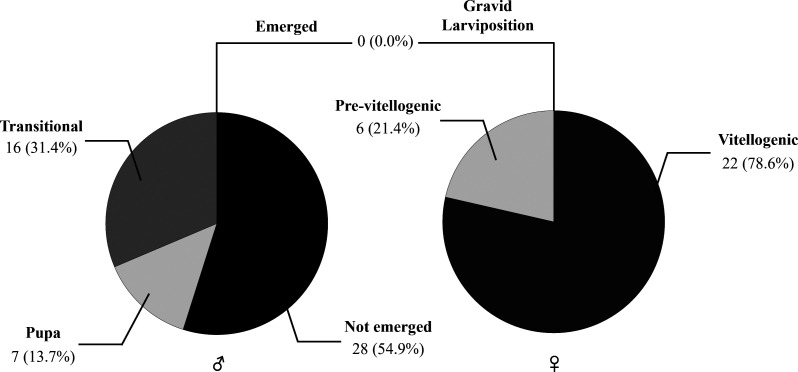
Sex-specific developmental stage composition of *X. oxyodontes* in stylopized *V. analis* individuals collected from 22 nests.

**Figure 7:**
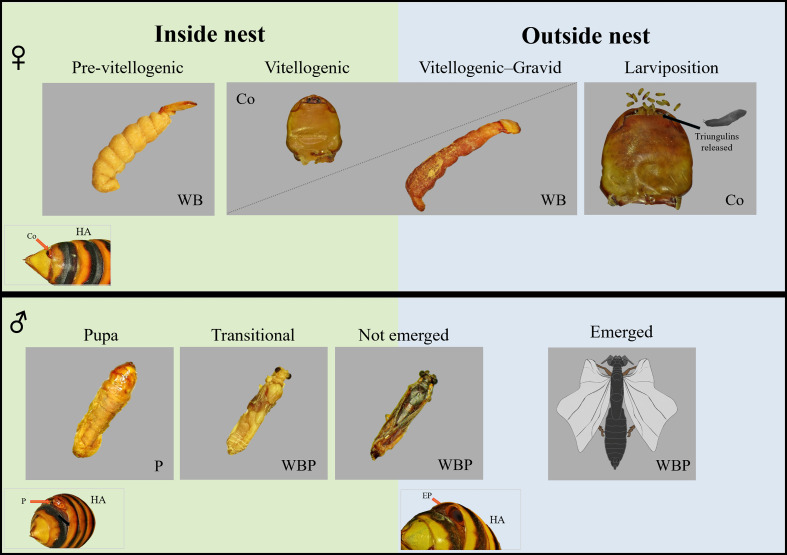
Representative developmental stages of *Xenos oxyodontes* recovered from inside and outside *Vespa analis* nests. Vitellogenic females and not-emerged males were detected in both inside- and outside-nest samples, suggesting that nest departure may occur at or around these stages. Abbreviations: WB, whole body (female); Co, cephalothorax (female); HA, host abdomen; EP, empty puparium (male); P, puparium (male); WBP, whole body in puparium (male).

Across the 22 nests, the occurrence of stylopized hornets showed substantial among-nest variation, with no stylopized hornet detected in 31.82% of the nests, whereas stylopized hornets were confirmed in many (Fig. 5). A total of 79 *X. oxyodontes* individuals were recorded, comprising 28 females and 51 males (Fig. 6). Regarding developmental stages, *Xenos* males were mostly in puparium-associated stages (pupa, transitional, or not emerged), and no evidence of puparium emergence was observed. Females were in either the pre-vitellogenic or vitellogenic stage, whereas individuals in the gravid, larviposition, or post-larviposition stages were not detected (Figs. 6 and 7).

## Discussion

This study combined 15 years of trap data with nest dissection data from South Korea. We found that the seasonal timing of occurrence differed among reproductive stages in female *X. oxyodontes*, whereas stage-specific differences were not evident in *X. moutoni*, and that high expected stylopized-host counts per sampling frame tended to be more locally concentrated for *X. oxyodontes* than for *X. moutoni*. In the *V. analis* nests, mature reproductive stages were not detected among *Xenos*, with only pre-reproductive stages observed.

### Host range and detection probability

Our trap results showed that expected stylopization tended to be spatially non-uniform for both *Xenos* species, with stronger local concentrations for *X. oxyodontes* than for *X. moutoni*, suggesting that stylopization by either species may be greater in specific regions or amplified under certain conditions. However, such concentration could be exaggerated by differences in host availability, habitat- or host-use patterns, or trap-based detection probabilities, and should therefore be validated by pairing nest-based sampling with trap data within the same localities [11]. Although nest-based surveys have reported that *V. velutina*, like *V. simillima*, can be stylopized by both *X. oxyodontes* and *X. moutoni* [15], no stylopized *V. velutina* individuals were detected in our trap samples, suggesting that differences between nest-based and trap-based sampling frameworks strongly influence observed patterns [11].

### Stage-specific phenology and extranidal emergence

The time of year in which different reproductive stages of female *X. oxyodontes* were encountered in trapped *Vespa* wasps differed. In contrast, *X. moutoni* showed no clear stage-specific seasonal differences, with stages occurring in a relatively uniform pattern. This may suggest that transitions among developmental stages in *X. oxyodontes* are concentrated within specific seasonal periods. Larvipositing-stage female *X. oxyodontes* individuals found in the host during late autumn would be expected to have invaded host eggs produced within the same year, suggesting these individuals may represent a second or later generation, arising from parasitism that occurred during the midseason. Interestingly, a phenology study on this species in Japan proposed that individuals detected in nests during the late season represent the second generation of the year, and the authors argued for a bivoltine cycle in this species [23]. Similar logic has been considered for the European species *Xenos vesparum* Rossi, 1793, for which Beani *et al.* (2018) [3] reported a bivoltine cycle in *Polistes* wasps in Italy, with female parasites releasing larvae twice, once in spring and once in summer. Therefore, the seasonal distribution of *X. oxyodontes* in South Korea should also be interpreted with the possibility of a bivoltine or multivoltine life cycle in mind [19].

The absence of evidence for male emergence and the lack of reproductive stage females in our nest samples are consistent with the possibility that some key reproductive events occur outside the nest. Importantly, in *V. analis* wasps collected using traps, male puparia have been reported to be largely absent, raising the possibility that hosts leave the nest before male emergence, such that emergence occurs outside the nest. Similarly, Makino (2001) [21] hypothesized, based on trap-based observations, that mating may not be restricted to the interior of the host’s nest. Strepsipterans have a life history in which the female remains within the host’s body, and because dispersal to new hosts depends on triungulin transfer, host movements are repeatedly discussed as being linked to transmission success [12, 13]. In the *Polistes*–*Xenos* association system in particular, field observations and experiments have shown that stylopized hosts cease colony tasks, leave the nest, and aggregate externally, and it is believed that these aggregation sites may coincide with mating, larval release, and overwintering [2, 10]. As males are extremely short-lived, it is especially important that females attract them via pheromones during their brief lifespan, and reproductive maturity is, thus, expected to be synchronized between the sexes [5, 8, 12]. This context makes our findings even more intriguing, raising further questions about the timing of mating in *X. oxyodontes*. However, because our nest dissections were based on a single early-September time point, the present data alone cannot distinguish between whether mature individuals had already departed or simply whether pre-mature stages predominated at the time of sampling.

While late-season larviposition was documented in our trap records, no mature reproductive stages were detected in the early-September nest dissections, making it difficult to conclude that the life cycle is completed entirely within the host’s nest. Although triungulins are often treated as a key element in discussions of voltinism, it remains necessary to determine whether triungulins released in the September–October period can proceed through overwintering. Notably, prior empirical studies have repeatedly supported the idea that stylopization prolongs host survival, allowing stylopized hosts to survive into the following year, and the strepsipteran subsequently larviposits [4, 33]. Accordingly, our data are not simply a reiteration of an extranidal scenario; the central implication is that there is a need to determine which pathways, such as host overwintering and extended longevity, allow late-season larviposition or triungulin release to connect to the next season, *i.e.*, what mechanisms link successive generations [21].

For *X. moutoni*, however, given that worker-caste hosts are unlikely to survive for extended periods in the way queens do, it is difficult to explain how a cohort produced in late-season (September–October) larviposition could overwinter successfully and resume activity the following spring. The observation of larvipositing-stage females in September–October, and the fact that these cases were primarily associated with worker hosts, suggests that – given the known constraints on the worker lifespan – larvae released within the same year may have the potential to carry over into the next generation. Thus, a multivoltine life history may be common in Korean populations, but because our study does not include direct individual tracking, we present bi- or multi-voltinism only conservatively here, at the level of plausibility. This line of inference could be strengthened in future work by combining concurrent sampling at extranidal aggregation and overwintering sites with season-long series of nest samples.

## Conclusion

In this study, we combined 15 years of trap data from South Korea with dissections of *V. analis* nests to provide a baseline synthesis of the phenology and stylopization patterns of two xenids, *X. oxyodontes* and *X. moutoni*. In the trap-based analyses, *X. oxyodontes* showed a tendency for the occurrence of female reproductive stages to vary seasonally, whereas stage-specific differences were limited in *X. moutoni*, and the spatial distributions of the two species were not equivalent within the shared sampling frame. Specifically, the concentration of high host-capture–adjusted expected stylopized-host counts was more localized in *X. oxyodontes* than in *X. moutoni*. In contrast, no mature reproductive-stage *Xenos* individuals were collected from *V. analis* nests in an early September sampling, with only pre-mature stages found, raising the possibility that some key reproductive events occur outside the nest or that late-season larviposition detected in trapped hosts and within-nest stage compositions may be temporally and/or spatially decoupled. Accordingly, rather than asserting voltinism, our data sharpen the research agenda by highlighting the need for a better understanding of how late-season larviposition and triungulin release are linked to the subsequent season. Future work that couples trap and nest sampling within the same localities and incorporates season-long series of nest samples could elucidate this link.

## Supplementary material

*Supplementary file*: Geographic coordinates and annual deployment information for the 677 trap sites used in this study. The supplementary material of this article is available at https://www.parasite-journal.org/10.1051/parasite/2026028/olm.
